# Ultrasound-Guided Forearm Nerve Blocks: A Novel Application for Pain Control in Adult Patients with Digit Injuries

**DOI:** 10.1155/2016/2518596

**Published:** 2016-07-31

**Authors:** Richard Amini, Parisa Patricia Javedani, Albert Amini, Srikar Adhikari

**Affiliations:** ^1^Department of Emergency Medicine, Tucson, AZ, USA; ^2^Arizona Premier Surgery, Chandler, AZ, USA

## Abstract

Phalanx fractures and interphalangeal joint dislocations commonly present to the emergency department. Although these orthopedic injuries are not complex, the four-point digital block used for anesthesia during the reduction can be painful. Additionally, cases requiring prolonged manipulation or consultation for adequate reduction may require repeat blockade. This case series reports four patients presenting after mechanical injuries resulting in phalanx fracture or interphalangeal joint dislocations. These patients received an ultrasound-guided peripheral nerve block of the forearm with successful subsequent reduction. To our knowledge, use of ultrasound-guided peripheral nerve blocks of the forearm for anesthesia in reduction of upper extremity digit injuries in adult patients in the emergency department setting has not been described before.

## 1. Introduction

Upper extremity digit injuries are a common orthopedic injury managed by emergency department (ED) physicians. Standard management is a prereduction X-ray, four-point digital nerve block, manipulation and reduction, and a postreduction X-ray. In prolonged dislocations, difficult manipulations, fractures, or cases requiring consultation, repeat blockade may be necessary. Alternative methods of analgesia and anesthesia include procedural sedation and regional nerve blocks. Procedural sedation requires monitoring during and after the procedure, places increased demands on staff, has a number of associated risks, increases ED length of stay, and is generally unnecessary for adequate reduction [1, 2].

Prior to the widespread use of ultrasound by emergency medicine (EM) physicians, nerve block use was largely limited to anesthesiologists due to the risk for hematoma formation, inadequate anesthesia, and potential for vascular infiltration [3]. Ultrasound guidance minimizes these risks, and trained EM providers are using nerve blocks with increasing frequency. Use of peripheral nerve blocks of the forearm in the ED has been reported for a number of indications including complex volar lacerations of the hand, carpal fracture reductions, and metacarpal fracture reductions [4, 5]. This case series presents four patients with phalanx fractures or dislocations who underwent peripheral nerve blocks of the forearm with successful and painless reduction.

## 2. Case Report

### 2.1. Case  1

A 30-year-old right hand (RH) dominant Hispanic male with no past medical history presented to the ED after injuring his finger during a recreational football game. The patient attempted to catch the ball but jammed and dislocated the fourth digit of his RH at the middle phalangeal joint (MPJ). The patient presented with the following vital signs: blood pressure (BP) 126/83 mmHg, heart rate (HR) 90 beats/min, respiratory rate (RR) 18 breaths/min, SpO_2_ 100% on room air (RA), and temperature 36.3°C. The patient underwent a hand radiograph that demonstrated a fracture dislocation of the proximal phalanx. The patient received an ultrasound-guided ulnar nerve block using a total of 5 mL of 1% lidocaine without epinephrine for pain control. After five minutes, adequate anesthesia was obtained as noted by the patient, and the reduction and splinting were successfully performed. The ED physician noted the reduction was easy to perform; the patient noted only minimal pain while performing the nerve block and was pain-free during the reduction and splinting of the digit. The patient had complete return of sensation to his hand prior to discharge. Vital signs at the time of discharge were blood pressure (BP) 120/80 mmHg, heart rate (HR) 80 beats/min, respiratory rate (RR) 20 breaths/min, and SpO_2_ 100% on room air (RA). The patient was discharged with a prescription for Vicodin for pain control.

### 2.2. Case  2

19-year-old RH dominant male presented to the ER complaining of hand pain after trying to jump onto a moving train approximately twenty hours prior to presentation. The patient was unable to flex the third digit at the MPJ. The patient's vital signs were as follows: BP 140/78 mmHg, HR 71 beats/min, RR 18 breaths/min, SpO_2_ 99% on room air, and temperature 36.8°C. The patient had an abrasion over the third proximal interphalangeal joint (PIP) without associated laceration or open injury. The patient demonstrated tenderness to palpation over the second and third PIP joints. X-ray demonstrated subluxation of the third digit, middle phalanx. An ultrasound-guided nerve block of the median nerve was performed, using an in-plane approach, to administer a total of 6 mL of 1% lidocaine without epinephrine. Complete anesthesia as noted by the patient was achieved one minute after completing the median nerve block. The ED physician was able to achieve reduction with ease; the patient felt minimal pain associated with the nerve block and manipulation associated with the reduction was painless. The patient had complete return of sensation to his hand prior to discharge and his pain was 1 out of 10. The patient was able to flex and extend his third digit across all joints. His vital signs upon discharge were BP 123/75 mmHg, HR 74 beats/min, RR 18 breaths/min, and SpO_2_ 100% RA.

### 2.3. Case  3

**Figure 1:**
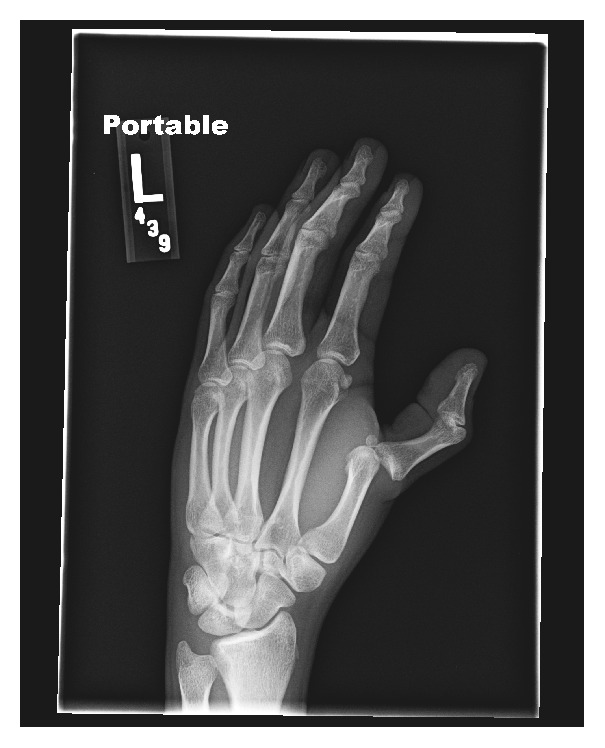
Radiograph demonstrating dislocated metacarpal-phalangeal joint of the first digit.

20-year-old RH dominant male presented to the ER with a left hand deformity that occurred while playing football. He reported pain on palpation of the metacarpophalangeal (MCP) joint of the left thumb with decreased sensation of the left thumb. The patient's vital signs were as follows: BP 127/62 mmHg, HR 74 beats/min, RR 18 breaths/min, SpO_2_ 100% on RA, and temperature 36.8°C; pain was reported as 10/10. The patient's hand radiograph showed dorsal dislocation of the left thumb at the MCP joint without evidence of fracture (Figure 1). The patient underwent an in-plane ultrasound-guided radial and median peripheral nerve block using a total of 12 mL of 1% lidocaine with epinephrine and after two minutes the reduction procedure was performed. The patient tolerated the procedure well; the left MCP joint was successfully reduced and his finger was splinted. The patient had complete return of sensation to his hand, was able to flex, extend, abduct, and oppose his thumb, and noted that his pain was 0/10 prior to discharge. Vital signs at the time of discharge were BP 118/49, HR 80 beats/min, RR 20 breaths/min, and SpO_2_ 100% on RA. He was given prescription for ibuprofen and Vicodin at the time of discharge.

### 2.4. Case  4

**Figure 2:**
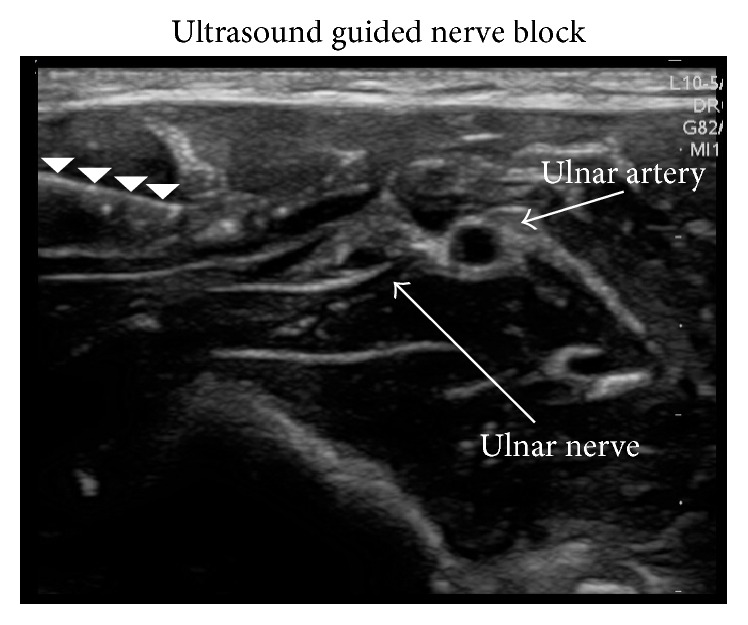
Ultrasound image demonstrating needle and anesthetic solution around the ulnar nerve.

25-year-old left hand dominant female presented to the ED after an all-terrain vehicle accident. She reported tenderness with palpation of the left fifth metacarpal but had normal sensation and two-point discrimination. The patient's vital signs were as follows: BP 110/76 mmHg, HR 93 beats/min, RR 16 breaths/min, SpO_2_ 98% on RA, and temperature 36.9°C; pain was reported as 10/10. The patient's hand radiograph showed a comminuted and dorsally displaced fracture of the left fifth metacarpal. The patient underwent an in-plane left ulnar nerve block under ultrasound guidance (Figure 2). The fracture was successfully reduced, the injury was splinted, and pain was noted to be 4/10. At the time of discharge, vitals were BP 102/51, HR 75, RR 16, and SpO_2_ 100% on RA, and the patient was given a prescription for Vicodin for pain control at the time of discharge.

## 3. Discussion

Phalanx fractures and interphalangeal joint dislocations are common injuries that arise from a number of mechanisms. ED physicians use ultrasound-guided peripheral nerve blocks to provide pain control for various procedures involving the hand, primarily for complex laceration repairs, and digit fracture reductions [5]. Ultrasound-guided forearm nerve blocks have also been investigated in children, and one study reported 5 of 10 patients in their convenience sample involved digit dislocation reductions [4]. To our knowledge, there are no reported case series of using an ultrasound-guided peripheral nerve block for phalanx fractures and interphalangeal joint dislocation reductions in adult patients in the ED. This series reports four cases utilizing an ultrasound-guided peripheral nerve block using 1% lidocaine with or without epinephrine to successfully reduce phalanx fractures and dislocated interphalangeal joint. Nerve distributions are highlighted in Table 1. The anesthetic used was left at the discretion of the treating physician, but it is likely that 1% lidocaine was selected due to its quick onset of action, decreased pain at the time of injection, and more than adequate duration of action to successfully complete the reduction procedure [6]. Additionally, the limited duration of action was advantageous, as return of distal sensation was confirmed prior to discharge; patients were discharged home with adequate pain medication.

In our case series, the ultrasound-guided nerve blocks were very well tolerated by all four patients. All reported minimal pain while the peripheral nerve block was performed, and none of the patients reported pain during the reduction. Furthermore, all patients had return of sensation prior to discharge. In our experience, the pain perceived by patients during ultrasound-guided nerve blocks appears to be much less when compared to patients receiving digital nerve blocks. Discriminatory sensations and nerve sensitivity increase in distal upper extremities, which may explain why patients perceive increased pain during the digital nerve blocks. Furthermore, ultrasound-guided nerve blocks are performed away from the injury site, which appears to have improved tolerability by patients. Finally, the use of ultrasound for nerve blockade provides distraction as patients are drawn to the ultrasound monitor, distracted during the procedure, and may perceive diminished pain during the nerve block procedure.

Our case series suggests that the ED physician can easily perform ultrasound-guided peripheral nerve blocks for pain control in patients requiring manipulation and reduction of digit dislocations and fracture-dislocations. The patients in this report obtained complete anesthesia within 5 minutes of performing the ultrasound-guided nerve block and obtained complete return of sensation prior to discharge. This is a shorter time to onset compared to studies in which ultrasound-guided forearm blocks were used to provide anesthesia to the hand [5]. None of the patients in this report required rescue anesthesia during the procedure, similar to findings reported in the literature [5]. No adverse events occurred with the procedure. The time necessary to prepare for the procedure is minimal. Although this procedure requires using the ultrasound machine, a peripheral forearm block anesthetizes one larger nerve whereas the traditional digital nerve block involves four smaller nerves. Furthermore, real-time ultrasound guidance allows direct visualization of underlying vascular structures and direct visualization of the nerve increasing the likelihood of complete anesthesia (Figure 2). It also does not cause distortion of the tissue caused by direct injection of anesthetic into the affected site. While peripheral nerve blocks have been widely studied in the ambulatory surgical setting, we are not aware of any reported case series using ultrasound-guided peripheral nerve blocks for phalanx fractures and interphalangeal joint dislocation reductions in adult patients presenting to the ED.

## 4. Conclusion

Upper extremity digit fractures and dislocations are commonly reduced with the traditional four-point digital block. An alternative method, ultrasound-guided peripheral nerve blocks of the forearm, is relatively easy to learn and has a quick time to onset of nerve blockade. Additionally, these blocks are minimally painful for the patient, make the reduction less difficult, and provide longer duration of effect for dislocations where prolonged manipulation or consultation may be required.

## Figures and Tables

**Table 1:** 

Nerve	Dorsal surface	Palmar surface
Radial nerve	Radial aspect of the dorsal surface of 1st through 3rd digits proximal to the distal interphalangeal joint; radial surface of the dorsum of the 4th digit proximal to the distal interphalangeal joint	

Median nerve	Dorsal fingertips including the distal interphalangeal joint	Palmar surface including the 1st–3rd digits and the radial palmar surface of the 4th digit

Ulnar nerve	Ulnar aspect of the dorsal surface of the hand including the dorsal ulnar aspect of the 4th digit and dorsal surface 5th digit; hypothenar eminence	Palmar surface including the ulnar of the 4th digit and palmar surface 5th digit
